# Gustave Roussy Immune Score (GRIm-Score) as a Prognostic and Predictive Score in Metastatic Colorectal Cancer

**DOI:** 10.7759/cureus.58935

**Published:** 2024-04-24

**Authors:** Horia Cotan, Cristian Iaciu, Emilescu Radu, Tudor Niculae, Oana A Rosu, Cornelia Nitipir

**Affiliations:** 1 Oncology, Carol Davila University of Medicine and Pharmacy, Bucharest, ROU; 2 Gastroenterology, Carol Davila University of Medicine and Pharmacy, Bucharest, ROU; 3 Nephrology, Nephrology Hospital Dr. Carol Davila, Bucharest, ROU; 4 Oncology, Elias Emergency University Hospital, Bucharest, ROU

**Keywords:** neutrophil-to-lymphocyte ratio (nlr), tumor microenviroment, inflammatory prognostic score, prognostic score, metastatic colorectal cancer

## Abstract

Introduction: Metastatic colorectal cancer (mCRC) presents significant clinical challenges due to its heterogeneous nature and variable treatment responses. The Gustave Roussy Immune Score (GRIm-Score) has emerged as a potential biomarker for prognostication and prediction in mCRC, although its precise role remains under investigation.

Methods: We conducted a retrospective study that included 173 patients diagnosed with mCRC. The patients were treated in the first line with 5-fluorouracil (5-FU)-based chemotherapy (CHT) and a molecular agent based on their eligibility. We assessed the overall survival (OS) time, progression-free survival (PFS) time, and the overall response rate (ORR), utilizing the GRIm-score measured at baseline (referred to as GRImT0) and the variance between GRImT0 and the GRIm score measured three months after treatment initiation (referred to as GRIm∆). We also performed a subgroup analysis based on the type of treatment received.

Results: Our analysis revealed that the GRIm-Score holds promise as a prognostic marker in mCRC, with high scores correlating with poorer survival outcomes. However, in the subgroup analysis, this prognostic value remained relevant only for patients treated with CHT and anti-EGFR (epidermal growth factor receptor) agents, such as cetuximab and panitumumab. GRIm-Score exhibited no predictive value irrespective of the treatment received.

Conclusion: The GRIm-Score shows potential as a prognostic mCRC, although we believe that this potential is limited. Integration of the GRIm-Score into clinical practice should be done with caution and is not recommended at this time. However, further research is needed to fully elucidate its clinical utility and optimize its incorporation into routine clinical care.

## Introduction

Colorectal cancer (CRC) stands as a considerable global health challenge, particularly with metastatic forms presenting significant hurdles in clinical care and patient prognosis. As of 2020, CRC ranks as the third most commonly diagnosed cancer worldwide, trailing behind breast and lung cancers, with a staggering 1,931,590 cases reported. Alarming statistics reveal that CRC accounted for 9.4% of all cancer-related deaths in the same year, totaling 935,173 fatalities. Projections indicate a worrying trend, with anticipated increases to 3.2 million new cases and 1.6 million deaths annually by the year 2040 [1,2]. Despite significant advancements in surgical techniques and medical care strategies, the long-term prognosis for individuals with CRC still presents opportunities for improvement. While early detection methods such as screening programs have contributed to better outcomes by enabling earlier intervention [3], CRC can still be challenging to treat effectively, especially in cases where the cancer has metastasized or recurred. Although at least three primary molecular pathways can result in CRC [4], the genetic composition of tumor cells alone is insufficient for subclassifying tumor types or reliably forecasting patient survival. Colon carcinogenesis serves as a prime illustration that tumor evolution relies not only on the existence of numerous critical mutations but also on the intimate interaction between mutagenized cells and their tumor microenvironment (TME) [5]. The role of TME in CRC carcinogenesis and prognosis has been well researched [6], and consequently, numerous pathological [7,8] and clinical scoring scores [9-12] have been developed and validated. Among the emerging biomarkers, the Gustave Roussy Immune Score (GRIm-Score) has been elaborated by Bigot et al. [13] as a substitute for the Royal Marsden Hospital (RMH) prognostic score in the selection of patients undergoing treatment with immune-checkpoint therapies (ICTs) during phase I trials.

The GRIm-Score is established upon serum lactate dehydrogenase (LDH), neutrophil-to-lymphocyte ratio (NLR), and serum albumin levels, demonstrating substantial prognostic value linked with the overall survival (OS) of cancer patients. The prognostic relevance of preoperative GRIm-Score has been reported in several retrospective studies in various cancer types, such as early-stage non-small-cell lung cancer [14-16], small-cell lung cancer [17], esophageal squamous cell carcinoma [18], CRC [18], and pancreatic cancer [19].

Although the GRIm-Score has been identified as an independent prognostic factor in various studies, its potential as a predictive biomarker for patients with CRC has yet to be assessed. This retrospective study seeks to determine the correlation between the GRIm-Score and the therapy response rate in patients with metastatic colorectal cancer (mCRC).

## Materials and methods

Study population

All patients involved in this retrospective study received treatment and follow-up at the Oncology Department of Elias Emergency University Hospital in Bucharest, Romania, from January 2016 to January 2024. All participants in this study were diagnosed with stage IV CRC. Treatment for these patients primarily involved palliative chemotherapy (CHT), with options including doublet CHT (FOLFOX (folinic acid (leucovorin, FOL), fluorouracil (5-FU, F), and oxaliplatin (OX)), FOLFIRI (folinic acid (leucovorin, FOL), fluorouracil (5-FU, F), and irinotecan (IRI)), or CAPEOX (capecitabine (CAPE) and oxaliplatin (OX))) or triplet CHT (FOLFOXIRI (folinic acid (leucovorin, FOL), fluorouracil (5-FU, F), oxaliplatin (OX), and irinotecan (IRI))), combined with the administration of bevacizumab. For patients with left-sided, RAS/BRAF wild-type tumors, a treatment regimen consisting of doublet CHT (FOLFOX, FOLFIRI, or CAPEOX) and an anti-EGFR agent (cetuximab or panitumumab) was administered. Notably, none of the stage IV patients included in this study received immunotherapy treatment.

The inclusion criteria required a confirmed positive diagnosis of CRC through histopathological and immunohistochemical assessments, along with precise clinical staging. Pathological staging was conducted by a seasoned pathologist utilizing the American Joint Committee on Cancer (AJCC) TNM Staging Classification for Colon Cancer 8th edition, 2017. Clinical staging involved comprehensive CT or MRI scans of the chest, abdomen, and pelvis, along with brain MRI scans for symptomatic patients. Lesions suspected to be metastases but not definitively identified as such on imaging underwent biopsy for histopathological and immunohistochemical confirmation. Exclusion criteria encompassed the presence of any indicators or symptoms suggestive of infection, such as elevated procalcitonin levels, leukocytosis, fever, malaise, abnormal chest radiography, or positive cultures from blood, urine, or pharyngeal exudate, as these factors could potentially affect the final results. Patients with immunocompromised status or autoimmune pathologies were also excluded. In addition, individuals undergoing corticosteroid therapy were excluded from the study. Patients diagnosed with other synchronous cancers were similarly excluded.

**Figure 1:**
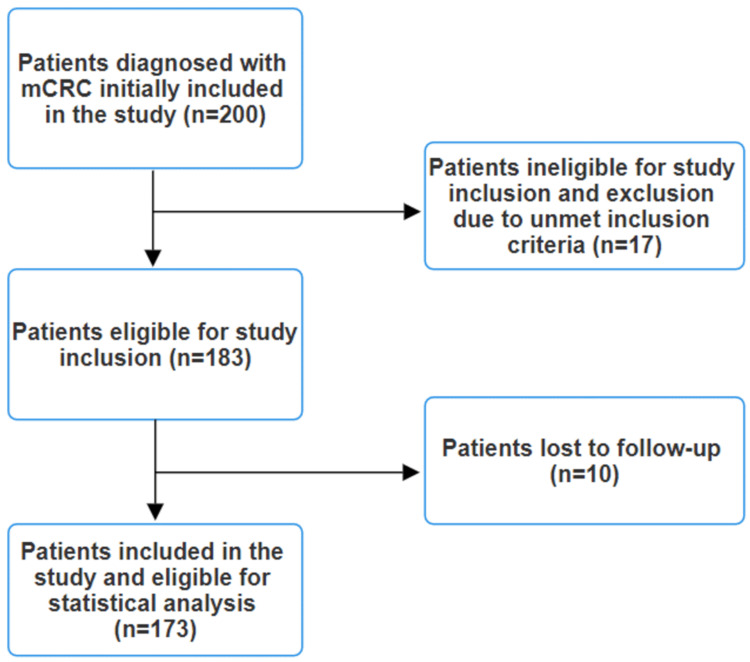
Study flow diagram

Initially, 200 patients with mCRC were included in the study. However, after applying the exclusion criteria, a total of 173 patients remained eligible for inclusion in the study (Figure 1).

Data collection

We conducted retrospective data collection encompassing demographic information (age, sex, and family history), tumor characteristics (location, differentiation, TNM stage, and molecular biomarkers), treatment modalities, and laboratory parameters (complete blood count, serum albumin, lactate dehydrogenase (LDH), liver function tests, carcinoembryonic antigen (CEA), and carbohydrate antigen 19-9 (CA19-9)).

**Figure 2:**
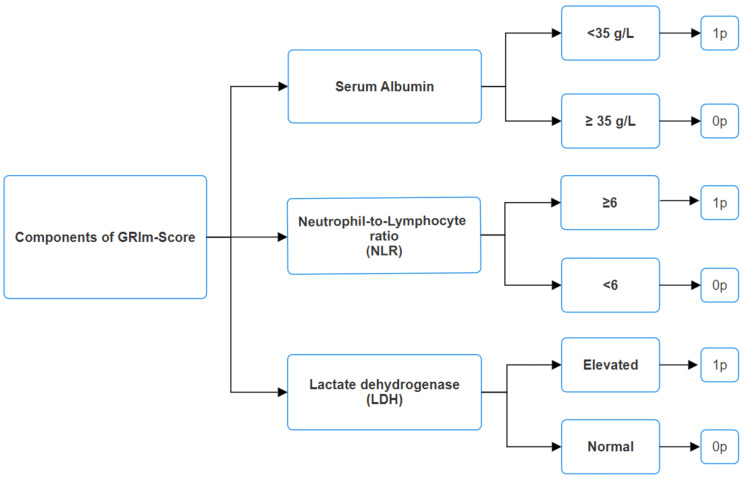
Components of the Gustave Roussy Immune Score (GRIm-Score)

The GRIm-Score was determined following the methodology outlined by Bigot et al. [13], which relies on three biomarkers: neutrophil-to-lymphocyte ratio (NLR), lactate dehydrogenase (LDH) levels, and serum albumin concentration. Patients were assigned a score of 1 if they exhibited any of the following criteria: NLR > 6, LDH levels exceeding the upper limit of normal, or serum albumin < 3.5 g/dL (Figure 2). This resulted in a total possible score of 3. A GRIm-Score below 2 was classified as a low score.

We also assessed the GRIm-Score at two distinct time points: baseline (GRImT0), which occurred within three days preceding the initial infusion, and three months following treatment initiation (GRImT1); the variation between these two time points was noted as GRIm∆ (GRImT0-GRImT1). A stable or positive GRIm∆ indicated that the GRIm-Score remained unchanged or decreased by at least one point, while a negative GRIm∆ indicated an increase in GRIm-Score between baseline and GRImT1.

Statistical analysis

Statistical analysis was performed using IBM SPSS Statistics for Windows, Version 26.0 (released 2019, IBM Corp., Armonk, NY). Patient and disease characteristics were reported using descriptive statistics. Differences between groups were assessed using Pearson's Chi-squared test or Fisher's exact test for categorical variables, while the two-sample T-test or Wilcoxon rank-sum test was employed for continuous variables. The median survival time was evaluated using the Kaplan-Meier method. To compare survival distributions between the two groups, the log-rank test was employed, providing insights into any significant differences in survival outcomes. Both univariate and multivariate Cox regression models were utilized to explore the relationship between elevated levels of GRIm-Score and the risk of death or recurrence among patients with CRC. These regression models allowed for the depiction of associations while adjusting for potential confounding variables. OS was calculated from the time of diagnosis to the date of death. Progression-free survival (PFS) was calculated from the initiation of treatment until the first documented progression, as per RECIST criteria. The overall response rate (ORR) was defined as the proportion of patients achieving either partial or complete response to the first line of therapy. We categorized partial response, complete response, and stable disease as favorable responses, while progressive disease was considered unfavorable.

Continuous variables with a normal distribution were expressed as mean and standard deviation (SD), whereas those without a normal distribution were presented as median and quartiles. Categorical variables were presented as counts (n) and percentages (%). Results with a p-value less than 0.05 were deemed statistically significant.

## Results

Patient characteristics

Out of 200 cases of mCRC patients treated with systemic therapy, only 173 were included in this analysis after the application of inclusion/exclusion criteria. There were 93 (53.8%) male patients and 80 (46.2%) female patients with an average age of 64.8 years with the range of 45-80 years old. Most patients (n = 143; 82.7%) were MSS/pMMR, while only 30 (17.3%) patients were MSI-H/dMMR. Treatment received varied depending on the RAS/BRAF mutational status and location of the primary tumor; most patients (n = 116; 67.1%) received doublet CHT and an anti-EGFR agent, while 20 (11.6%) and 37 (21.4%) received triplet CHT (FOLFOXIRI) and bevacizumab or doublet CHT (FOLFOX/CAPEOX/FOLFIRI) and bevacizumab, respectively. According to the GRIm-Score system, there were 71 (41%) patients who reached a score of 0 points, 44 (25.4%) patients who reached a score of 1, 40 (23.1%) patients who reached a score of 2, and 18 (10.4%) who reached a score of 3 at baseline (i.e., GRImT0). According to the GRIm score, after 60 days of treatment, there were 120 (69.4%) patients with low GRIm scores (0-1) and 53 (30.6%) patients with high GRIm scores (2-3).

The OS and PFS times were 24 months (95% CI 22.679-25.321, p = 0.030) and 14 months (95% CI 12.738-15.625), respectively. There was a statistically significant, but small difference in PFS (p = 0.004) between treatment groups was observed, with PFS for patients receiving doublet CHT and bevacizumab being 13 months (95% CI 11.626-21.167) and 14.5 months (95% CI 10.557-17.443) for those receiving triplet CHT and bevacizumab. The longest PFS was observed in patients treated with doublet CHT and an anti-EGFR agent, with a PFS time of 17 months (95% CI 10.531-23.469). Similarly, OS differed significantly (p = 0.001), with the longest survival seen in patients receiving doublet CHT and anti-EGFR agent, with an OS time of 29 months (95% CI 23.789-34.211), followed by those treated with triplet CHT and bevacizumab, with 24 months (95% CI 15.488-32.512). Patients treated with doublet CHT and bevacizumab had the shortest OS time of 22 months (95% CI 19.996-24.004). The information is depicted in Table 1.

**Table 1:** Baseline clinical characteristics of the patients. CI, confidence interval; PFS, progression-free survival; OS, overall survival; GRImT0, GRIm-Score at baseline; GRImT1, GRIm-Score measured after three months following treatment initiation; GRIm∆, GRIm-Score three months following treatment initiation; GRIm- GRIm-Score variation between the two time points (GRImT0 and GRImT1); ECOG, Eastern Cooperative Oncology Group; MSI-H/dMMR, microsatellite instability-high/deficient mismatch repair; MSS/pMMR, microsatellite stable/proficient mismatch repair; CEA, carcinoembryonic antigen; CA 19-9, cancer antigen 19-9

	Doublet CHT+ anti-EGFR agent (n = 116)	Triplet CHT + bevacizumab (n = 20)	Doublet CHT+ bevacizumab (n = 37)
Age, years, mean (SD)	65.4 (±5.43)	64.1 (± 6.22)	63.6 (± 7.42)
Gender, n (%)			
Male	65 (56%)	11 (55%)	17 (45.9%)
Female	51 (44%)	9 (45%)	20 (54.1%)
ECOG, n (%)			
0	64 (55.2%)	9 (45%)	15 (40.5%)
1	43 (37.1%)	2 (10%)	16 (43.2%)
≥ 2	9 (7.8%)	9 (45%)	6 (16.2%)
Primary tumor location, n (%)			
Right side	0 (0%)	8 (40%)	20 (54.1%)
Left side	116 (100%)	12 (60%)	17 (45.9%)
RAS mutational status, n (%)			
Wild-type	116 (100%)	12 (60%)	18 (48.6%)
Mutant	0 (0%)	8 (40%)	19 (51.4%)
BRAF mutational status, n (%)			
Wild-type	116 (100%)	16 (80%)	33 (89.2%)
Mutant	0 (0%)	4 (20%)	4 (10.8%)
MSI/MMR status, n (%)			
MSI-H/dMMR	27 (23.3%)	0 (0%)	3 (8.1%)
MSS/pMMR	89 (76.7%)	20 (100%)	34 (91.9%)
Tumor burden			
Extrahepatic extension	84 (72.4%)	12 (60%)	32 (86.5%)
Confined to the liver	32 (27.6%)	8 (40%)	5 (13.5%)
Resection of the primary tumor			
Primary tumor resected	8 (6.9%)	3 (15%)	2 (5.4%)
Primary tumor not resected	108 (93.1%)	17 (85%)	35 (94.6%)
Carcinoembryonic antigen (CEA)			
Over the upper limit	47 (40.5%)	9 (45%)	16 (43.2%)
Under the upper limit	69 (59.5%)	11(55%)	21 (56.8%)
Cancer antigen 19-9 (CA 19-9)			
Over the upper limit	15 (12.9%)	3 (15%)	0 (0%)
Under the upper limit	101 (87.1%)	17 (85%)	37 (100%)
GRImT0			
Low (0-1)	83 (71.6%)	10 (50%)	22 (59.4%)
High (2-3)	33 (28.4%)	10 (50%)	15 (40.6%)
GRImT1			
Low (0-1)	83 (71.6%)	11 (55%)	26 (70.3%)
High (2-3)	33 (28.4%)	9 (45%)	11 (29.7%)
GRIm∆			
Positive	21 (18.1%)	6 (30%)	10 (27%)
Stationary	79 (68.1%)	11 (55%)	24 (64.9%)
Negative	16 (13.8%)	3 (15%)	3 (8.1%)
Median PFS (months)	17 months (95% CI 10.531-23.469)	14.5 months (95% CI 10.557-17.443)	13 months (95% CI 11.626-21.167)
Median OS (months)	29 months (95% CI 23.789-34.211)	24 months (95% CI 15.488-32.512)	22 months (95% CI 19.996-24.004).

Clinical outcome according to GRImT0 and GRIm∆

**Figure 3:**
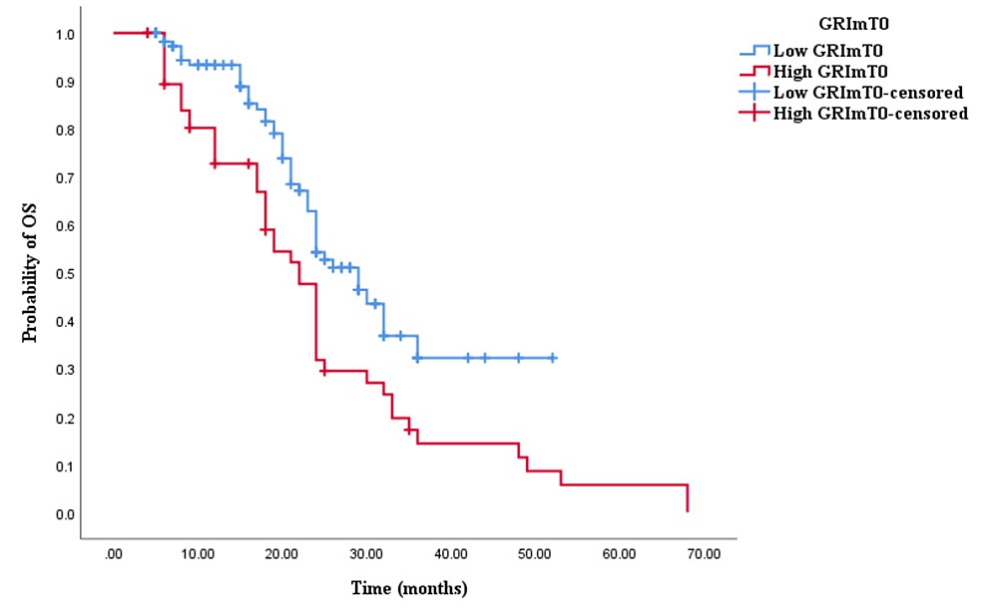
Comparative analysis of Kaplan–Meier curves between low GRImT0 and high GRImT0 patients for overall survival (OS).

**Figure 4:**
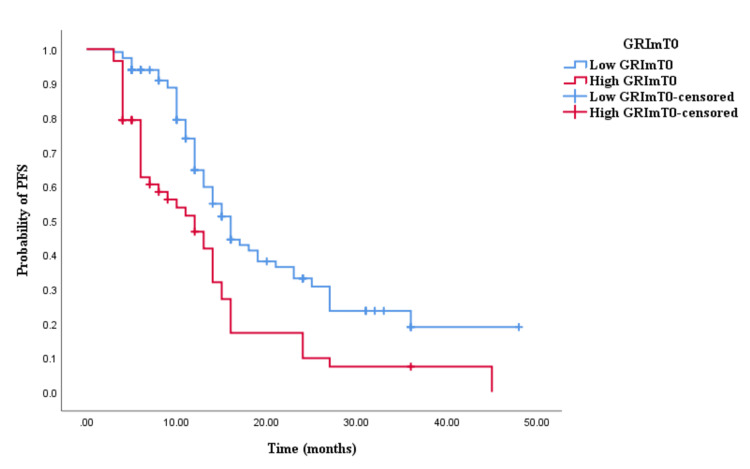
Comparative analysis of Kaplan–Meier curves between low GRImT0 and high GRImT0 patients for progression-free survival (PFS).

The Kaplan-Meier survival analysis revealed that CRC patients in the high GRImT0 group exhibited significantly worse OS with an OS time of 29 months (95% CI 24.645-33.355, p = 0.004; see Figure 3) compared to those in the low GRImT0 group with an OS time of 22 months (95% CI 18.926-25.074, p = 0.004; see Figure 3). Similarly, the survival analysis indicated that CRC patients with high GRImT0 experienced a shorter PFS time of 12 months (95% CI 8.033-15.967, p < 0.0001; see Figure 4) compared to those with low GRImT0, who exhibited a PFS time of 16 months (95% CI 13.928-18.072, p < 0.0001; see Figure 4).

**Figure 5:**
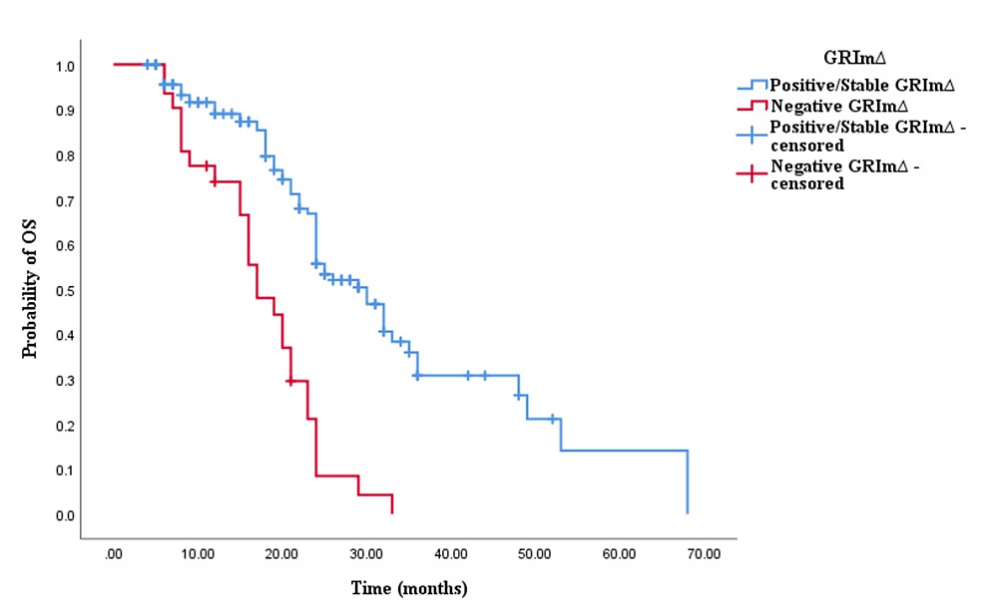
Comparative analysis of Kaplan–Meier curves between positive/stable GRIm∆ and negative GRIm∆ patients for overall survival (OS).

**Figure 6:**
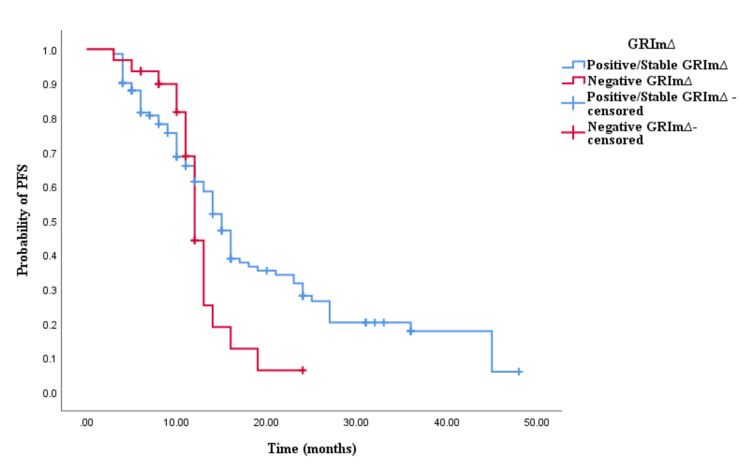
Comparative analysis of Kaplan–Meier curves between positive/stable GRIm∆ and negative GRIm∆ patients for progression-free survival (PFS).

Similarly, the Kaplan-Meier survival analysis demonstrated that CRC patients in the positive/stable GRIm∆ group exhibited significantly worse OS with an OS time of 30 months (95% CI 24.337-35.663, p < 0.0001; see Figure 5) compared to those in the negative GRIm∆ group with an OS time of 17 months (95% CI 12.001-21.999, p < 0.0001; see Figure 5). However, survival analysis did not reveal a significant difference in PFS time between CRC patients with positive/stable GRIm∆ and those with negative GRIm∆. Patients in the positive/stable GRIm∆ group had a PFS of 15 months (95% CI 8.033-15.967, p = 0.121; see Figure 6), while those with negative GRIm∆ exhibited a PFS time of 12 months (95% CI 13.928-18.072, p = 0.121; see Figure 6). 

Clinical outcome stratified by treatment according to GRImT0

**Figure 7:**
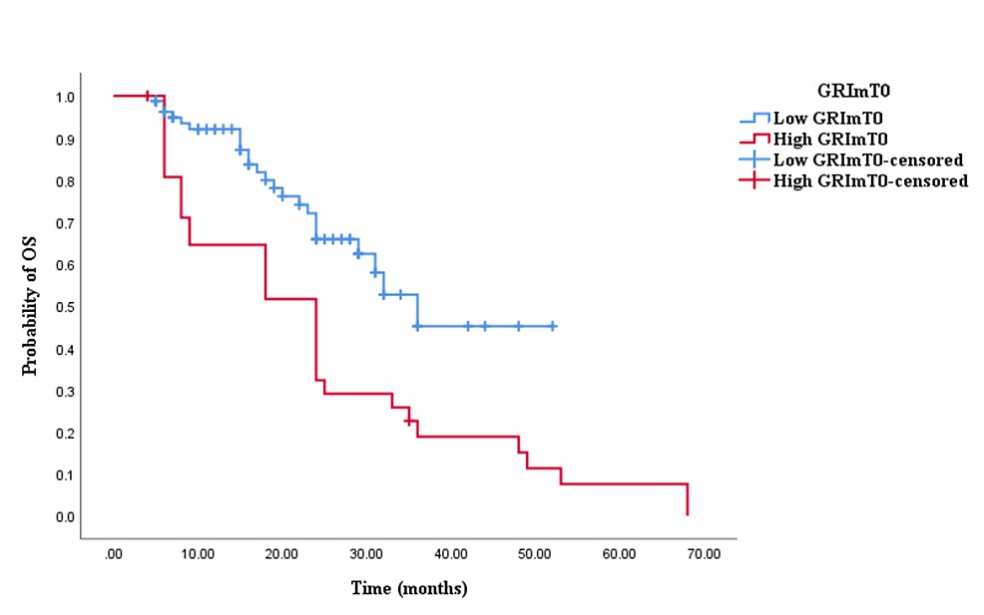
Comparative analysis of Kaplan–Meier curves between low GRImT0 and high GRImT0 patients for overall survival (OS) in the cohort treated with chemotherapy (CHT) doublet and bevacizumab

**Figure 8:**
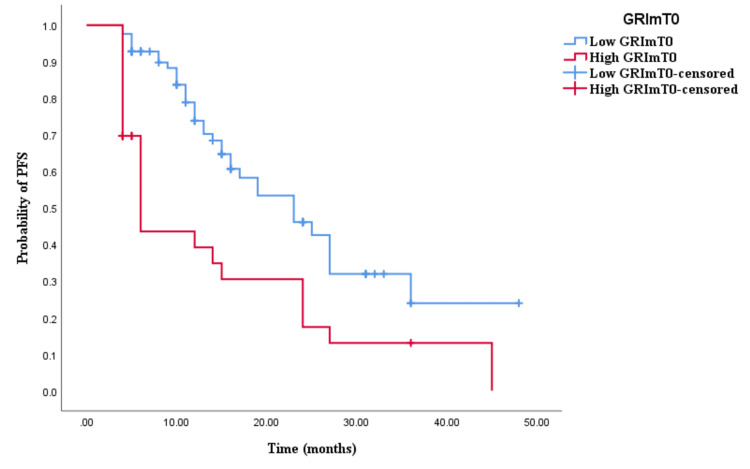
Comparative analysis of Kaplan–Meier curves between low GRImT0 and high GRImT0 patients for progression-free survival (PFS) in the cohort treated with chemotherapy (CHT) doublet and bevacizumab

**Figure 9:**
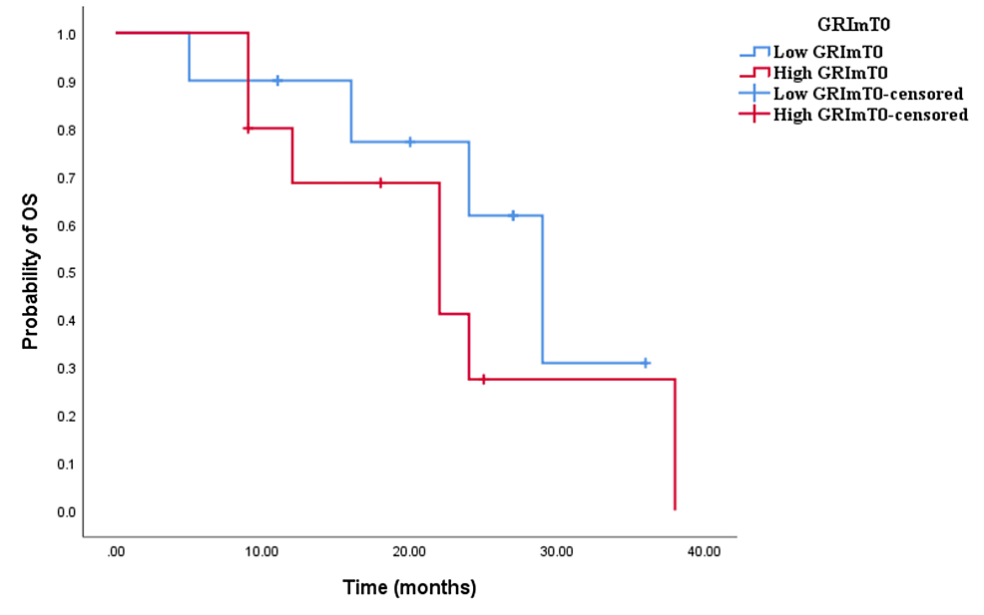
Comparative analysis of Kaplan–Meier curves between low GRImT0 and high GRImT0 patients for overall survival (OS) in the cohort treated with chemotherapy (CHT) triplet and bevacizumab

**Figure 10:**
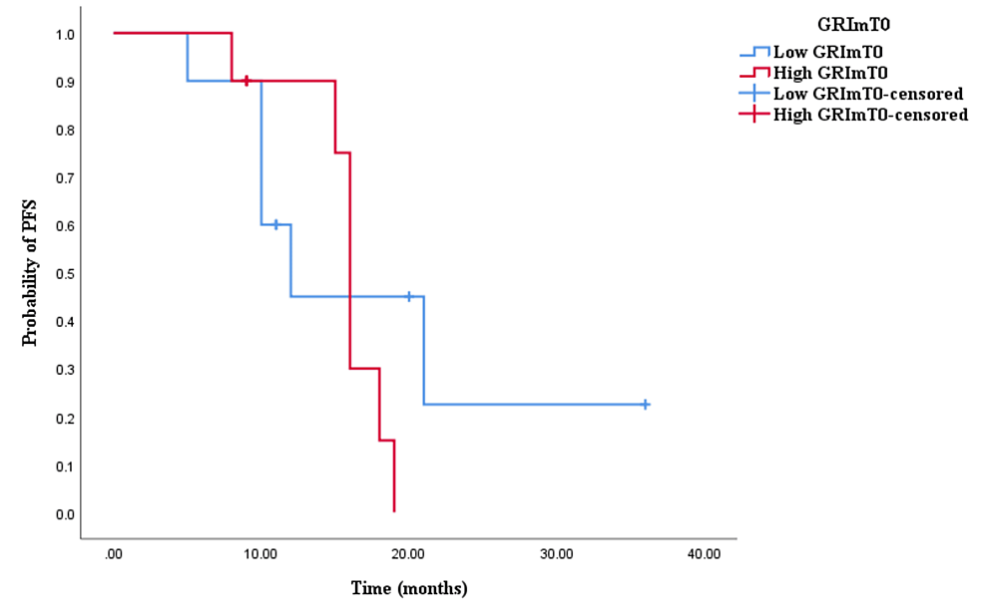
Comparative analysis of Kaplan–Meier curves between low GRImT0 and high GRImT0 patients for progression-free survival (PFS) in the cohort treated with chemotherapy (CHT) triplet and bevacizumab

In the cohort receiving doublet CHT and bevacizumab, a contrast in the distribution of low and high GRImT0 was noted, with 59.4% and 40.6%, respectively. However, there was no significant disparity observed between low and high GRImT0 scores concerning both OS and PFS (low vs. high: OS 23 vs. 22.2 months, p < 0.001; PFS 13 vs. 12 months, p < 0.001; see Figures 7, 8). Similarly, in the cohort treated with triplet CHT and bevacizumab, the proportion of low and high GRImT0 was comparable, at 50% each. Moreover, no notable difference was detected in clinical outcomes (low vs. high: OS 29 vs. 22 months, p = 0.127; PFS 12 vs. 16 months, p = 0.267; see Figures 9, 10).

**Figure 11:**
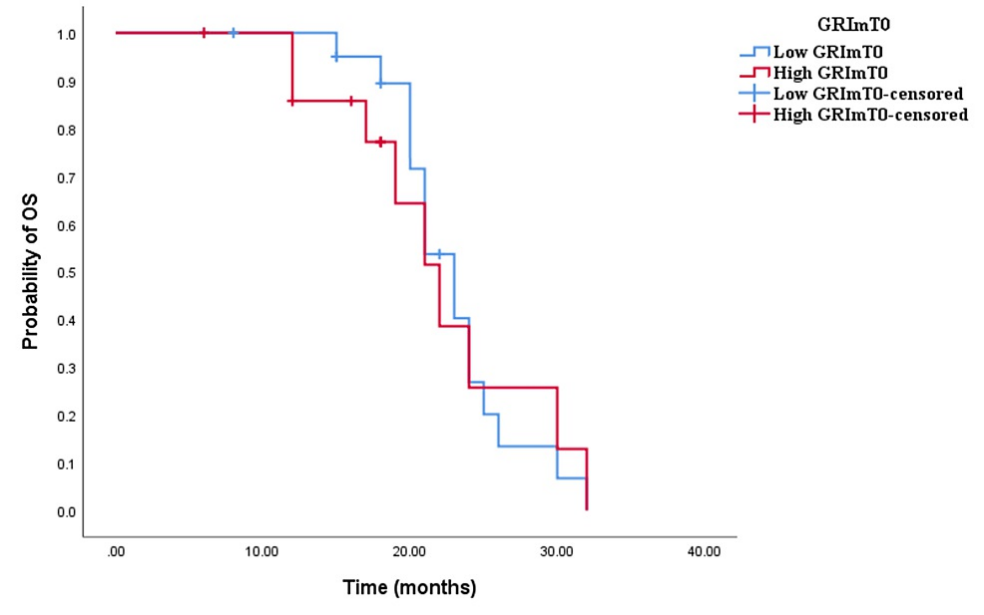
Comparative analysis of Kaplan–Meier curves between low GRImT0 and high GRImT0 patients for overall survival (OS) in the cohort treated with chemotherapy (CHT) doublet and anti-epidermal growth factor receptor (EGFR) agents (cetuximab/panitumumab)

**Figure 12:**
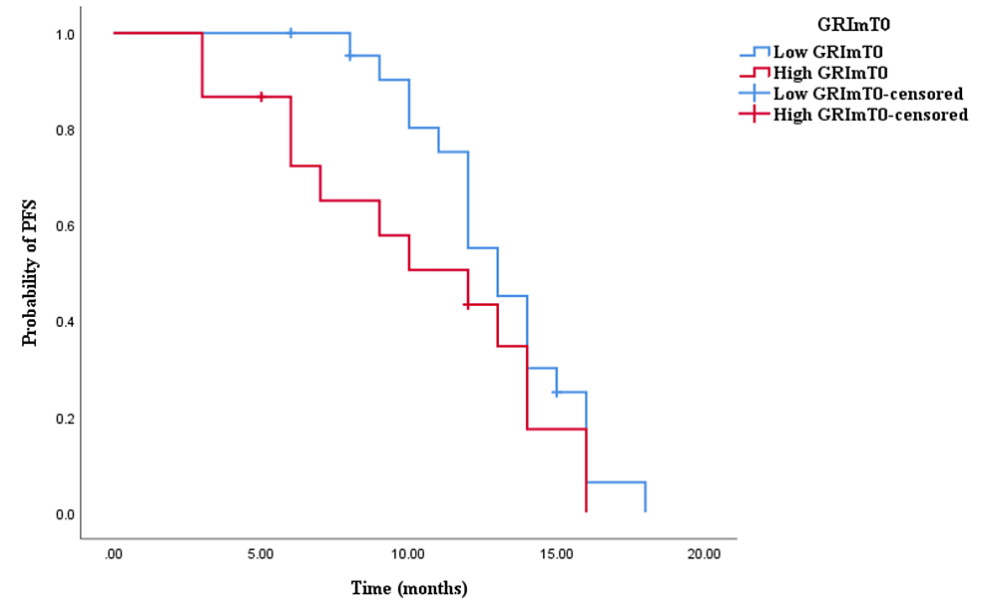
Comparative analysis of Kaplan–Meier curves between low GRImT0 and high GRImT0 patients for progression-free survival (PFS) in the cohort treated with chemotherapy (CHT) doublet and anti-epidermal growth factor receptor (EGFR) agents (cetuximab/panitumumab)

In the cohort receiving CHT doublet and an anti-EGFR agent, the majority of patients (71.6%) exhibited low GRImT0 scores, while the remainder (28.4%) had high GRImT0 scores. A significant contrast was found between patients with low and high GRImT0 scores in terms of both OS and PFS (low vs. high: OS 36 vs. 24.1 months, p < 0.001; PFS 23 vs. nine months, p = 0.002; see Figures 11, 12).

Clinical outcome stratified by treatment according to GRIm∆

**Figure 13:**
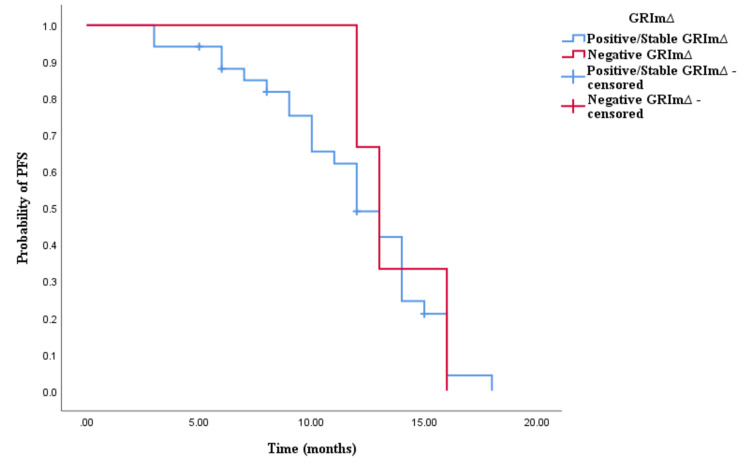
Comparative analysis of Kaplan–Meier curves between positive/stable GRIm∆ and negative GRIm∆ patients for progression-free survival (PFS) in the cohort treated with CHT doublet and bevacizumab

**Figure 14:**
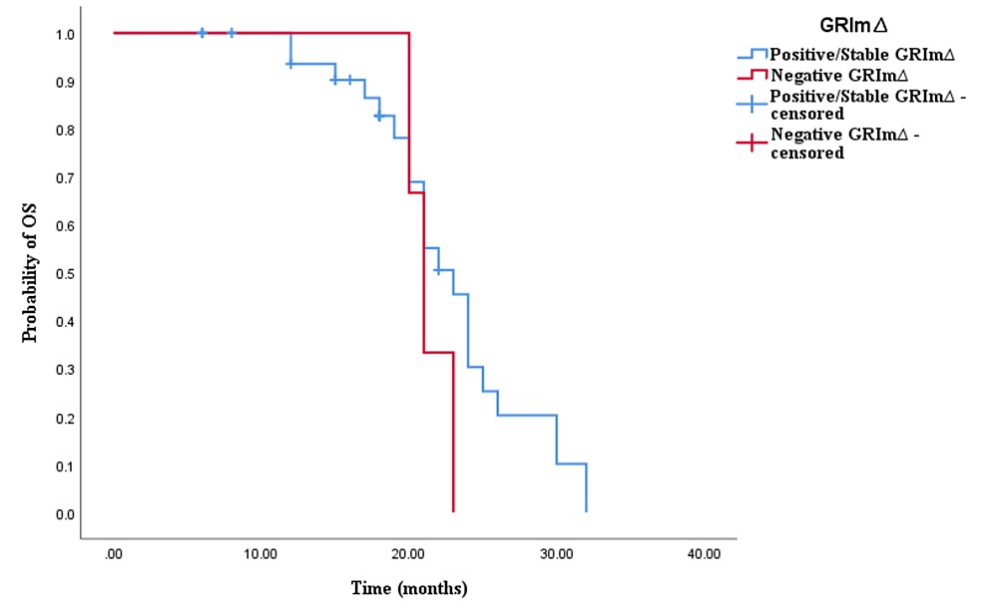
Comparative analysis of Kaplan–Meier curves between positive/stable GRIm∆ and negative GRIm∆ patients for overall survival (OS) in the cohort treated with chemotherapy (CHT) doublet and bevacizumab

**Figure 15:**
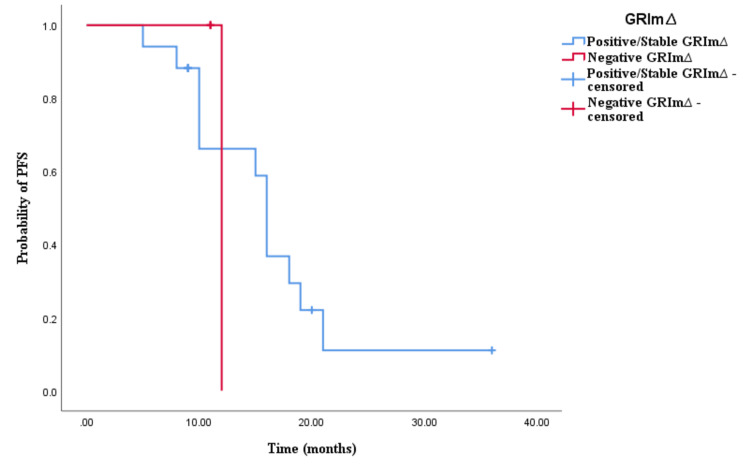
Comparative analysis of Kaplan–Meier curves between positive/stable GRIm∆ and negative GRIm∆ patients for progression-free survival (PFS) in the cohort treated with chemotherapy (CHT) triplet and bevacizumab

**Figure 16:**
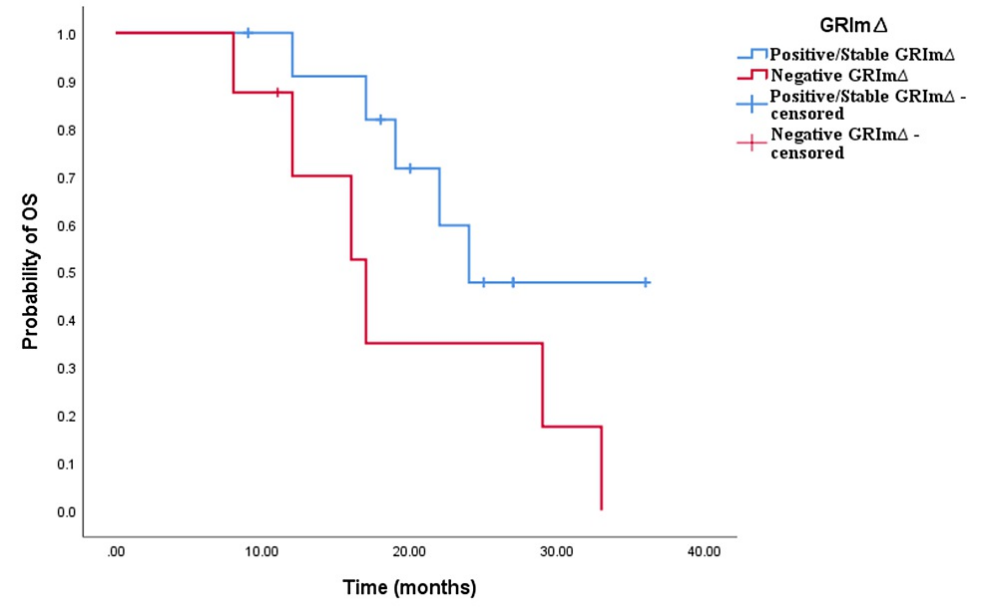
Comparative analysis of Kaplan–Meier curves between positive/stable GRIm∆ and negative GRIm∆ patients for overall survival (OS) in the cohort treated with chemotherapy (CHT) triplet and bevacizumab

**Figure 17:**
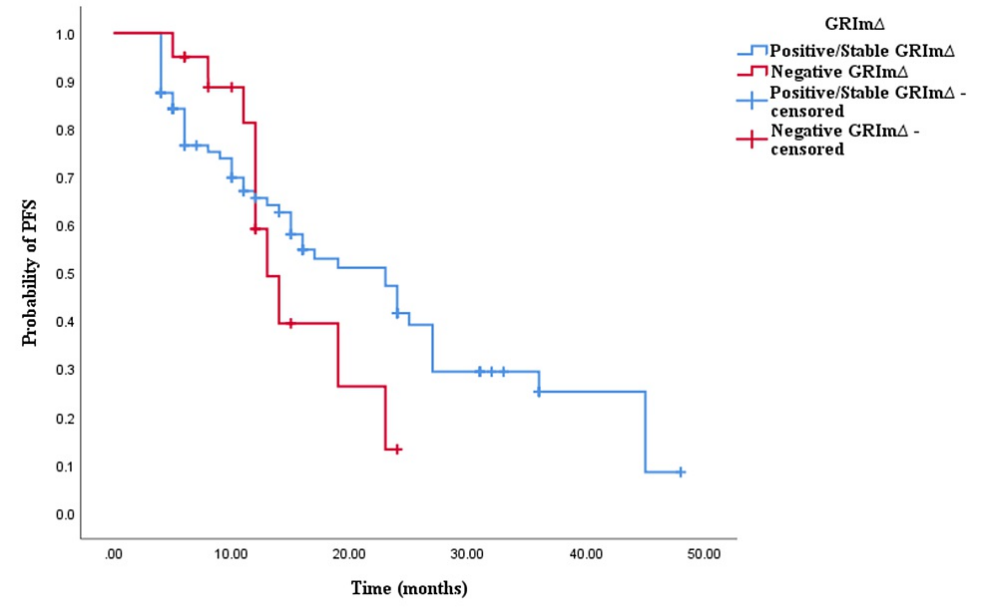
Comparative analysis of Kaplan–Meier curves between positive/stable GRIm∆ and negative GRIm∆ patients for progression-free survival (PFS) in the cohort treated with chemotherapy (CHT) doublet and anti-epidermal growth factor receptor (EGFR) agents (cetuximab/panitumumab)

**Figure 18:**
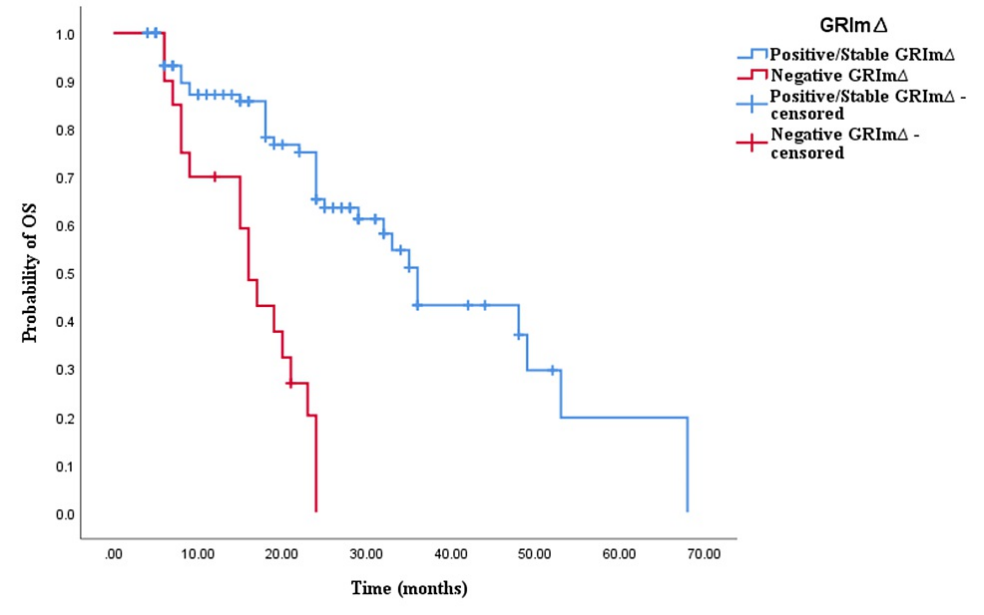
Comparative analysis of Kaplan–Meier curves between positive/stable GRIm∆ and negative GRIm∆ patients for overall survival (OS) in the cohort treated with chemotherapy (CHT) doublet and anti-epidermal growth factor receptor (EGFR) agents (cetuximab/panitumumab)

In the cohort receiving doublet CHT and bevacizumab, there was no significant disparity observed between positive/stable GRIm∆ and negative GRIm∆ concerning both OS and PFS (low vs. high: OS 23.5 vs. 21 months, p < 0.001; PFS 13 vs. 11.5 months, p < 0.001; see Figures 13, 14). Similarly, in the cohort treated with triplet CHT and bevacizumab, no notable difference was detected in clinical outcomes (GRIm∆ positive/stable vs. negative: OS 24 vs. 17 months, p = 0.095; PFS 16 vs. 12 months, p = 0.259; see Figures 15, 16). In the cohort receiving CHT doublet and an anti-EGFR agent, a significant contrast was found between patients with positive/stable GRIm∆ and negative GRIm∆ scores in terms of OS but not PFS (positive/stable vs. negative: OS 35 vs. 16 months, p < 0.001; PFS 23 vs. 13 months, p = 0.564; see Figures 17, 18).

Multivariate analysis in the CHT doublet and an anti-EGFR agent cohort

The prognostic and predictive value of GRImT0 and GRIm∆ have been confirmed in the CHT doublet and an anti-EGFR agent cohort through multivariate analysis (Cox regression). The hazard ratio (HR) for death among patients with a high GRImT0 was 4.091 (95% CI 2.241-7.469, p < 0.001; Table 2), while the HR for cancer progression was 4.799 (95% CI 2.762-8.338, p < 0.001; Table 2).

**Table 2:** Multivariate Cox regression analyses to identify predictors for increased risk of death and tumor progression considering GRImT0 as an independent variable CI, confidence interval; PFS, progression-free survival; HR, hazard ratio; OS, overall survival; GRImT0, GRIm-Score at baseline; ECOG, Eastern Cooperative Oncology Group; ref., reference; statistically significant (p < 0.05)

Test variables	OS HR (95% CI)	p-value	PFS HR (95% CI)	p-value
GRImT0 low (ref.)/high	4.091 (2.241-7.469)	<0.001	4.799 (2.762-8.338)	<0.001
BRAF mutational status wild-type (ref.)/ mutant	1.754 (0.984-2.604)	0.003	1.454 (0.984-2.604)	0.383
RAS mutational status wild-type (ref.)/ mutant	1.215 (0.568-1.863)	0.001	1.115 (0.568-1.863)	0.459
ECOG 0-1 (ref.)/2	1.490 (1.089-2.038)	0.013	1.102 (0.558-1.340)	0.087
Microsatellite instability MSI-H/dMMR (ref.)/MSS/pMMR	1.910 (0.852-4.282)	0.128	2.185 (1.865-6.722)	0.006
Tumor burden confined to the liver (ref.)/ extrahepatic extension	2.100 (0.923-5.125)	0.257	1.126 (0.672-3.725)	0.216
Carcinoembryonic antigen (CEA) low (ref.)/high	1.125 (0.682-3.249)	<0.001	1.245 (0.573-1.879)	0.003
Cancer antigen 19-9 (CA 19-9) low (ref.)/high	1.057 (0.655-1.729)	0.186	1.139 (0.763-2.456)	0.247
Age <65 years (ref.)/>65 years	1.390 (0.874-2.125)	0.238	1.198 (0.537-1.517)	0.698
Gender female (ref.)/male	1.447 (0.849-2.466)	0.174	1.338 (0.861-2.079)	0.195
Location of primary tumor left side (ref.)/ right side	1.120 (0.542-1.843)	0.454	1.423 (0.801-2.526)	0.229

Similarly, the HR for death among patients with a high GRIm∆ was 4.091 (95% CI 2.241-7.469, p < 0.001; Table 3), while the HR for cancer progression was 4.799 (95% CI 2.762-8.338, p < 0.001; Table 3).

**Table 3:** Multivariate Cox regression analyses to identify predictors for increased risk of death and tumor progression considering GRIm∆ as independent variable CI, confidence interval; PFS, progression-free survival; HR, hazard ratio; OS, overall survival; GRIm∆, GRIm- GRIm-Score variation between the two time points (GRImT0 and GRImT1); ECOG, Eastern Cooperative Oncology Group; ref., reference; statistically significant (p < 0.05)

Test variables	OS HR (95% CI)	p-value	PFS HR (95% CI)	p-value
GRIm∆ positive/stable (ref.)/negative	2.942 (1.694-5.109)	<0.001	1.750 (0.975-3.141)	0.005
BRAF mutational status wild-type (ref.)/ mutant	1.405 (0.640-3.087)	0.001	1.074 (0.508-2.272)	0.852
RAS mutational status wild-type (ref.)/ mutant	1.211 (0.494-1.724)	0.001	1.115 (0.568-1.863)	0.015
ECOG 0-1 (ref.)/2	1.802 (1.326-2.449)	0.001	0.970 (0.720-1.392)	0.490
Microsatellite instability MSI-H/dMMR (ref.)/MSS/pMMR	1.213 (0.570-2.579)	0.289	2.848 (1.667-4.865)	<0.001
Tumor burden confined to the liver (ref.)/ extrahepatic extension	1.978 (0.854-4.765)	0.416	1.126 (0.672-3.725)	0.216
Carcinoembryonic antigen (CEA) low (ref.)/high	1.361 (0.823-2.248)	0.019	1.115 (0.773-1.879)	0.003
Cancer antigen 19-9 (CA 19-9) low (ref.)/high	1.128 (0.655-1.860)	0.094	1.051 (0.663-1.666)	0.832
Age <65 years (ref.)/>65 years	1.261 (0.698-2.341)	0.244	1.104 (0.421-1.893)	0.776
Gender female (ref.)/male	1.175 (0.698-1.979)	0.542	1.213 (0.775-2.465)	0.245
Location of the primary tumor: left side (ref.)/ right side	1.415 (0.791-1.818)	0.544	1.225 (0.529-1.648)	0.229

We also examined the ORR categorized by GRImT0 score and GRIm∆. We have defined a favorable outcome as a partial response (PR), a complete response (CR), or a stable disease (SD) and an unfavorable outcome as a progressive disease (PD). The majority of low GRImT0 patients (n = 89, 77.4%; Table 4) showed a favorable response, whereas a smaller percentage of patients in the high GRImT0 group (n = 35, 60.3%; Table 4) exhibited a favorable response. A total of 96 patients (67.6%) with positive/stable GRIm∆ showed a favorable response, while paradoxically, a majority of GRIm∆ negative patients (n = 19, 86.4%; Table 4) also displayed a favorable response.

**Table 4:** Association between GRImT0, GRIm∆, and ORR ORR, overall response rate; GRIm∆, GRIm- GRIm-Score variation between the two-time points (GRImT0 and GRImT1); GRImT0, GRIm-Score at baseline; CR, complete response; PR, partial response; SD, stable disease; PD, progressive disease; statistically significant (p < 0.05)

ORR	Low GRImT0	High GRImT0	GRIm∆ positive/stable	GRIm∆. negative
Favorable response (CR+PR+SD) (n, %)	89 (77.4 %)	35 (60.3 %)	96 (67.6%)	19 (86.4 %)
Unfavorable response (PD) (n, %)	26 (22.6 %)	23 (39.7 %)	46 (32.4%)	3 (13.6 %)

We also conducted a univariate logistic regression analysis to evaluate the predictive significance of GRImT0 and GRIm∆ for ORR. However, neither GRImT0 nor GRIm∆ demonstrated prognostic value, yielding OR of 0.95 (95% CI 0.438-1.117, p = 0.10; Table 5) and 1.03 (95% CI 0.638-1.223, p = 0.07; Table 5), respectively.

**Table 5:** Univariate logistic regression analysis to assess the predictive value of GRImT0, GRIm∆ for ORR ORR, overall response rate; GRIm∆, GRIm- GRIm-Score variation between the two-time points (GRImT0 and GRImT1); GRImT0, GRIm-Score at baseline; ref., reference; CR, complete response; PR, partial response; SD, stable disease; PD, progressive disease; OR, odds ratio; statistically significant (p < 0.05)

Test variables	ORR OR (95% CI)	p-value
GRImT0 low (ref.)/high	0.95 (0.438-1.117)	0.10
GRIm∆ positive/stable (ref.)/negative	1.03 (0.638-1.223)	0.07

## Discussion

Inflammation within the tumor microenvironment has been recognized as a hallmark of cancer, contributing to tumor proliferation, angiogenesis, and immune evasion [20]. In CRC, chronic inflammation associated with conditions, such as inflammatory bowel disease (IBD) [21] or obesity [22], has been implicated in promoting tumorigenesis and influencing disease behavior. Moreover, inflammatory markers, such as C-reactive protein (CRP) [23], interleukin-6 (IL-6) [24], and tumor necrosis factor-alpha (TNF-α) [25], are elevated in CRC patients and correlated with tumor aggressiveness and poor prognosis.

One approach to quantifying the systemic inflammatory response in CRC patients is through the use of inflammatory scores, such as the Glasgow Prognostic Score (GPS) [26], NLR [27,12], platelet-to-lymphocyte ratio (PLR) [28], and modified Glasgow Prognostic Score (mGPS) [29]. These scores incorporate various peripheral blood parameters, including white blood cell counts, neutrophils, lymphocytes, and platelets, to stratify patients based on their inflammatory status.

In addition to prognostic implications, inflammatory markers and scores hold promise as predictive biomarkers for treatment response in CRC. Mounting evidence suggests that patients with high inflammatory scores may derive less benefit from standard therapies, such as chemotherapy [30,12], compared to those with low inflammatory scores. Incorporating inflammation-based biomarkers into patient selection algorithms could help identify individuals who are more likely to benefit from specific treatment modalities, thereby optimizing therapeutic efficacy and minimizing unnecessary toxicities.

The GRIm-Score as developed by Bigot et al. [13] evaluates both inflammatory and nutritional parameters, such as LDH, NLR, and serum albumin, with the main purpose of showcasing the systemic inflammation and nutritional status of CRC patients. This study represents the first comprehensive investigation into the role of both baseline GRIm-Score (GRImT0) and dynamic changes post-treatment initiation (GRIm∆) in mCRC patients. Encompassing a cohort of 173 patients receiving chemotherapy, our findings indicate that both GRImT0 and GRIm∆ emerge as independent prognostic factors solely among patients treated with a chemotherapy doublet (such as FOLFOX, FOLFIRI, or CAPEOX) in combination with an anti-EGFR agent (cetuximab or panitumumab). Notably, no prognostic significance was observed for patients receiving chemotherapy in conjunction with bevacizumab.

Furthermore, our analysis reveals that GRImT0 and GRIm∆ lack predictive value, as evidenced by similar response rates irrespective of the GRIm-Score's high or low value. This suggests that while GRIm-Score may provide valuable prognostic insights, it does not influence the likelihood of treatment response.

The analysis of the GRIm-Score in the context of CRC has been relatively limited, with only one prior retrospective study [18] examining similar aspects. Interestingly, our study yielded comparable results regarding the predictive and prognostic value of the GRIm-Score. However, notable distinctions exist between our study and the aforementioned one.

First, the previous study focused on patients who had undergone treatment with curative intent and had received adjuvant therapy, while our investigation concentrated on patients with mCRC undergoing chemotherapy associated with molecular therapy (anti-VEGF or EGFR agents).

Second, in the previous study, the GRIm-Score was identified as an independent prognostic factor specifically among CRC patients treated with chemotherapy or observation alone. This contrasts with our findings, where, in a subgroup analysis, the GRIm-Score demonstrated prognostic significance only within the subgroup of patients receiving chemotherapy combined with an anti-EGFR agent.

There are several possible reasons for this phenomenon. First, the addition of bevacizumab to chemotherapy has not been shown to improve OS in patients with mCRC, as demonstrated by the phase III NO16966 trial [31]. In this 1400-patient study, CAPEOX (capecitabine dose of 1000 mg/m^2^ administered twice daily for 14 days) combined with either bevacizumab or placebo was compared with FOLFOX combined with bevacizumab or placebo. The addition of bevacizumab to oxaliplatin-based regimens led to a modest increase of 1.4 months in PFS compared to these regimens without bevacizumab (hazard ratio (HR) 0.83; 97.5% CI 0.72-0.95; p = 0.0023). However, the observed difference in OS, also 1.4 months, did not reach statistical significance (HR 0.89; 97.5% CI 0.76-1.03; p = 0.077). Several hypotheses have been proposed regarding the variations observed when comparing NO16966 with other trials. These differences may stem from variances in treatment discontinuation rates and durations; however, these hypotheses remain speculative. Nevertheless, in this randomized study, there was no discernible difference in response rate with or without bevacizumab, indicating that early withdrawal rates would not have influenced this outcome. Subset analyses within the study assessing the benefit of adding bevacizumab to either FOLFOX or CAPEOX indicated that while bevacizumab was associated with improvements in PFS when added to CAPEOX, this benefit was not observed with FOLFOX.

An integral component of the GRIm-Score is the NLR. As previously noted, NLR has been extensively investigated, albeit retrospectively, in CRC. A subgroup analysis of the TRIBE trial [32] has revealed the prognostic significance of NLR in mCRC patients treated with bevacizumab plus chemotherapy in the first line, highlighting the poorer prognosis of patients with high NLR. Importantly, the benefit of the triplet regimen remains independent of NLR at baseline. Similarly, in our study, FOLFOXIRI and bevacizumab treatment was superior in terms of OS and PFS compared to doublet CHT.

A meta-analysis [33] encompassing six randomized clinical trials (RCTs) involving 3,060 patients, assessing the efficacy of bevacizumab in first-line treatment for mCRC, demonstrated a notable advantage in PFS (HR 0.72; 95% CI 0.66-0.78; p < 0.00001) and OS (HR 0.84; 95% CI 0.77-0.91; p < 0.00001). However, subgroup analyses indicated that this advantage was predominantly observed in irinotecan-based regimens [34]. This meta-analysis may shed light on why no benefit in OS and PFS has been observed among patients with low GRImT0 or GRIm∆ treated with doublet CHT and bevacizumab. Our study did not differentiate between patients receiving irinotecan-based or oxaliplatin-based chemotherapy, potentially influencing the outcomes observed.

Cetuximab and panitumumab have been investigated as initial therapy options for the treatment of mCRC in combination with FOLFIRI and FOLFOX. The randomized, phase II PLANET-TTD trial [35], which compared patients treated with panitumumab plus either FOLFOX or FOLFIRI, found no significant differences in efficacy between the two regimens.

Meta-analyses of RCTs [36,37] have concluded that EGFR inhibitors provide a distinct clinical benefit in the treatment of patients with RAS wild-type mCRC.

The phase III PARADIGM trial [38] reported on the use of panitumumab versus bevacizumab when combined with FOLFOX as first-line therapy in 823 patients with RAS wild-type mCRC with left-sided tumors. After a median follow-up of 61 months, panitumumab demonstrated a significantly higher OS when used as a part of the first-line regimen compared to bevacizumab. This superiority in OS was observed in both the left-sided tumor population (37.9 vs. 34.3 months) and the full analysis set (36.3 vs. 31.3 months).

These studies suggest that the optimal therapeutic approach for mCRC patients eligible for anti-EGFR treatment involves combining chemotherapy with either cetuximab or panitumumab. In our study, patients treated with CHT and an anti-EGFR agent demonstrated superior OS and PFS compared to patients treated with bevacizumab and CHT, regardless of their GRImT0 or GRIm∆ status.

Our study aimed to evaluate the prognostic and predictive value of the GRIm-Score in mCRC. Overall, our findings suggest that the GRIm-Score holds some, albeit limited promise as a prognostic tool in this patient population, providing potential valuable insights into the immune microenvironment's influence on disease progression and treatment response. However, several limitations must be acknowledged to interpret our results cautiously.

First, our study relied on retrospective data from a single institution, which may introduce selection bias and limit the generalizability of our findings. The patient cohort was relatively small, and the retrospective nature of the analysis might have introduced confounding variables that were not adequately controlled for. Future prospective studies with larger, multicenter cohorts are warranted to validate our findings and confirm the utility of the GRIm-Score in diverse patient populations.

Second, although we assessed various clinicopathological factors, other potential confounders such as comorbidities, performance status, treatment compliance, and molecular profile were not comprehensively accounted for in our analysis. These factors may influence both the response rates and clinical outcomes in mCRC patients. Therefore, future studies should consider incorporating these variables to obtain a more comprehensive understanding of the GRIm-Score's prognostic and predictive value.

Third, our study focused solely on the GRIm-Score and its association with clinical outcomes in mCRC. While this score provides valuable information on the immune contexture within the tumor microenvironment, it does not capture other relevant biomarkers or molecular characteristics that may influence treatment response and prognosis. Integrating the GRIm-Score with other immune-related biomarkers or genomic signatures could potentially enhance its predictive accuracy and clinical utility.

Lastly, our study primarily assessed the prognostic value of the GRIm-Score in mCRC patients treated with standard chemotherapy regimens. The utility of the GRIm-Score in guiding treatment decisions, such as the selection of immunotherapy agents or combination therapies, remains to be elucidated. Future studies evaluating the predictive value of the GRIm-Score in the context of novel treatment modalities are warranted to optimize patient stratification and improve therapeutic outcomes.

## Conclusions

This study provides evidence that the GRIm-Score may have prognostic value in mCRC, albeit restricted to patients treated with cetuximab or panitumab in combination with 5-FU-based chemotherapy. However, we have not established the role of the GRImT0-Score as a predictive marker. Our findings suggest that the role of the GRIm-Score is limited in therapy selection for mCRC. Currently, the optimal first-line chemotherapy for mCRC remains CHT combined with an anti-EGFR agent in eligible patients or FOLFOXIRI and bevacizumab in those ineligible for cetuximab/panitumumab. This notion is somewhat supported by our findings, indicating that regardless of the inflammatory score, the best survival outcomes were observed in cohorts receiving chemotherapy plus cetuximab/panitumumab.
